# Susceptibility to Beta-Cypermethrin, the F1534C Mutation, and MFO Amount in *Aedes aegypti* from Dengue-Endemic Areas of Yunnan Province, China, in 2015–2016

**DOI:** 10.3390/insects17060573

**Published:** 2026-05-31

**Authors:** Qing-Ming Shi, Qin-Mei Liu, Ai-Juan Sun, Chun-Xiao Li, Xiao-Xia Guo, Dan Xing, Tong-Yan Zhao, Heng-Duan Zhang

**Affiliations:** 1 State Key Laboratory of Pathogen and Biosecurity, Beijing 100071, China; qingmings2026@163.com (Q.-M.S.); qmliu@cdc.zj.cn (Q.-M.L.); xmk@szcdc.cn (A.-J.S.); vectorlcx@126.com (C.-X.L.); guoxx99@163.com (X.-X.G.); xingdan93@163.com (D.X.); tongyanzhao@126.com (T.-Y.Z.); 2 Center for Disease Control and Prevention in Western Theater Command, Lanzhou 730030, China; 3 Zhejiang Provincial Center for Disease Control and Prevention, Hangzhou 310051, China

**Keywords:** *Aedes aegypti*, pyrethroid resistance, dengue fever, VGSC gene mutation, biochemical assays

## Abstract

*Aedes aegypti* is a major invasive vector in China, where prolonged pyrethroid use has induced resistance, complicating dengue control. This study evaluated the resistance levels of *Ae. aegypti* to the pyrethroid beta-cypermethrin in Yunnan and explored the underlying mechanisms to inform control strategies. Mosquitoes were collected from five regions of Yunnan in 2015–2016. Larval bioassays, adult diagnostic dose determination, and adult bioassays were conducted to assess resistance to beta-cypermethrin. Mixed-function oxidase (MFO) amount was measured in larvae, and PCR amplification was used to detect mutations in the voltage-gated sodium channel (VGSC) gene. Correlations between enzyme activity, mutations, and phenotypic resistance were analyzed. Larval bioassays indicated that all five populations exhibited resistance to beta-cypermethrin, with resistance ratios ranging from 11.31 to 41.56. The adult diagnostic dose was determined as 0.74668 g/L, with mortality rates ranging from 8.89% to 58.89%, confirming resistance. A significant negative correlation was found between adult mortality and larval LC_50_ values. MFO amount was closely correlated with resistance levels. The F1534C mutation was the only VGSC mutation detected, and its frequency showed a significant positive correlation with beta-cypermethrin resistance. Our data were collected in 2015–2016; resistance levels may have changed since then, and no confirmatory bioassays were performed by us after 2020. The findings demonstrate that the F1534C mutation and increased MFO amount contribute to enhanced beta-cypermethrin resistance of *Ae. aegypti* in Yunnan. By linking phenotype resistance to key molecular and biochemical mechanisms, these findings support the continued monitoring of resistance and provide a basis for the evidence-based optimization of dengue vector control strategies.

## Simple Summary

This study investigated beta-cypermethrin phenotype resistance, metabolic resistance, and target resistance in *Ae. aegypti* collected from five dengue-endemic areas in Yunnan Province, China, during 2015–2016. Larval and adult bioassays confirmed moderate to high resistance to beta-cypermethrin in all field populations. Biochemical assays revealed an elevated mixed-function oxidase (MFO) amount, which was correlated with resistance levels. Molecular screening identified the F1534C mutation in the voltage-gated sodium channel (VGSC) gene as the sole knockdown resistance (kdr) mutation, and its frequency was significantly positively correlated with beta-cypermethrin resistance. These results demonstrate that the F1534C mutation and increased MFO amount together confer pyrethroid beta-cypermethrin resistance to *Ae. aegypti* in Yunnan. Our data provide a resistance baseline for dengue vector control and support the need for continuous resistance monitoring and mechanistic research to improve vector management strategies.

## 1. Introduction

*Aedes aegypti* is the major vector of some important arboviruses, such as dengue fever and Zika, which are distributed throughout tropical and subtropical areas worldwide, and in China, it is distributed mainly in Guangdong, Guangxi, Yunnan, and Hainan Provinces [1,2]. Recently, dengue has spread, affecting hundreds of millions of people worldwide [3]. Although the World Health Organization (WHO) has created many foundations to support research on the prevention and treatment of dengue fever and Zika, specific drugs are still unavailable, and the treatment is still restricted to supportive care [4,5]. Thus, prevention and control measures for dengue and chikungunya primarily focus on reducing mosquito densities, which is mainly accomplished through chemical approaches [6,7].

The use of insecticides to control the larvae or adults of vector mosquitoes is one of the preventive chemical measures against vector-borne diseases [8]. Pyrethroids are widely used for mosquito control because of their high efficiency, low toxicity, and relatively environmentally friendly properties [9,10]. The intensive and prolonged use of insecticides can select resistant specimens in natural vector populations, decreasing the frequency of susceptible individuals and increasing the variability of field populations [11]. *Ae. aegypti* has been reported to exhibit resistance to various pyrethroid insecticides, and resistance is on the rise; this situation has created some problems in vector control programs in many countries [12,13,14,15].

Pyrethroids induce a characteristic intoxication known as the knockdown effect, in which the insect undergoes fast and repetitive muscle spasms followed by paralysis and eventual death [16]. One of the main mechanisms underlying resistance to these classes of insecticides is target gene mutation, the voltage-gated sodium channel (VGSC) gene mutation. Currently, the VGSC gene mutations related to pyrethroid insecticides that have been identified in *Ae. aegypti* mosquitoes include V410L, G923V, L982W, I1011M, I1011V, V1016G, S989P, F1534C, T1520I, and D1763Y [17,18,19,20,21,22].

Metabolic resistance is another major physiological mechanism and refers to an increase in the synthesis of detoxifying enzymes or in their ability to metabolize the insecticide, both of which result in an increase in the detoxifying capacity of the insect [23]. Glutathione S-transferases (GSTs), carboxylesterases (ESTs), and multifunction oxidases (MFOs, also known as P450s) are the key classes of enzymes generally involved in this process, and all of these enzymes belong to families composed of several genes [13,24,25]. Among these detoxifying enzymes, the MFO class deserves attention in the resistance of *Ae. aegypti* to pyrethroids, as revealed by high-throughput assays that compared the overall profile at the genomic and transcriptome levels between resistant populations and susceptible strains [26].

As an important invasive vector mosquito in China, breeding *Ae. aegypti* was first detected in Yunnan in 2002 [27]. Since its discovery, pyrethroid insecticides have been extensively used to prevent its reinvasion and expansion, as well as to control the spread of the disease carried by this species, which results in high levels of resistance to pyrethroids [19]. Determining the susceptibility and related resistance mechanisms of *Ae. aegypti* to insecticides commonly used for mosquito control is urgently needed to implement effective and sustainable arbovirus vector control measures. We therefore examined the distribution and insecticide susceptibility of both larval and adult *Ae. aegypti* at five sites in Yunnan Province and screened the possible detoxification enzyme activities and VGSC gene mutations of these *Ae. aegypti* populations to clarify the potential resistance mechanisms.

## 2. Materials and Methods

### 2.1. Mosquito Sampling and Rearing

**Figure 1:**
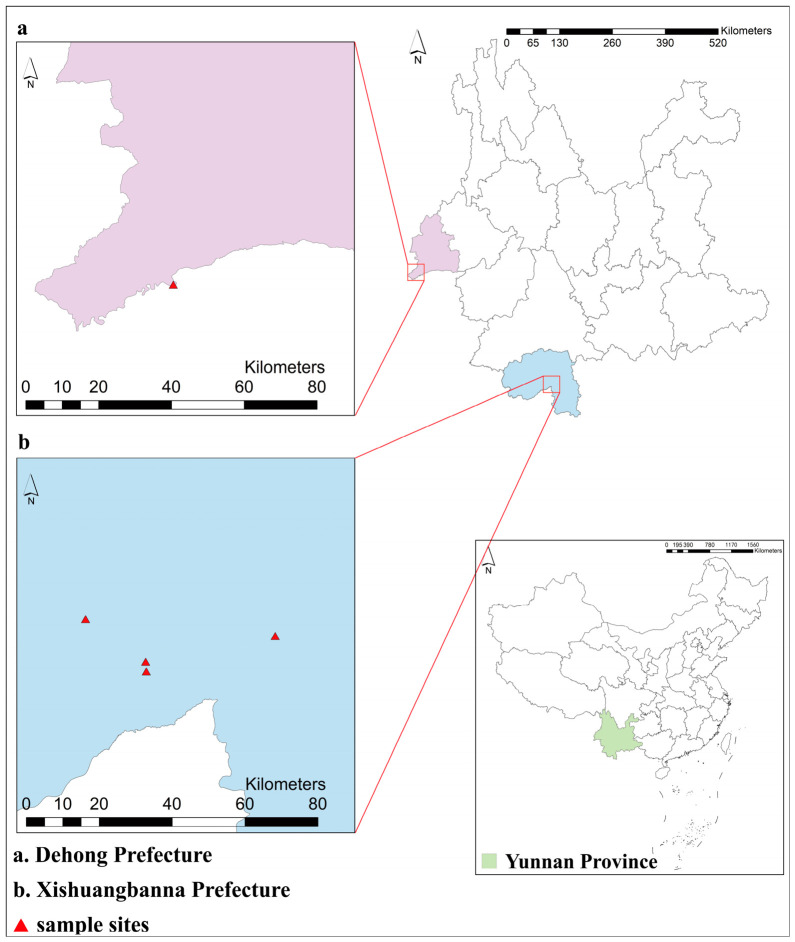
Map of collection sites in Dehong and Xishuangbanna of Yunnan Province, China. The triangles correspond to different collection sites.

*Aedes aegypti* were sampled as larvae or pupae at five locations in Yunnan Province (Table 1, Figure 1) from June to September 2015 in Dehong Prefecture and from May to July 2016 in Xishuangbanna Prefecture. Immature stages (field generation, F0) were collected from water in domestic (e.g., jars and tanks), peridomestic (e.g., tires), and natural environments (e.g., tree holes). For each sampling site, larvae or pupae from about 10 larval breeding locations were collected, stored in plastic boxes, and transferred to insectaries for rearing to the adult stage. After the F_0_ generation was morphologically identified as *Ae. aegypti* [28], each population (more than 200 females) was reared to the F1 generation for larval and adult bioassays. All mosquitoes were maintained under standard insectary conditions at 26 ± 1 °C, 65% ± 10% relative humidity, a 10:14 h light/dark photoperiod, and supplementation with powdered fish food (0.1 g per 100 larvae, once daily) for larvae until they developed into pupae, and 8% glucose solution for adult *Ae. aegypti* from Yunnan Province, which was maintained in the laboratory and had not been exposed to insecticides for ten years, was used as a sensitive control strain (Nruili 2004).

### 2.2. Larval and Adult Bioassays

The susceptibility of the larvae to beta-cypermethrin was assessed using the sensitive baseline method outlined by the GB/T 26347-2010 protocol [29]. Stock solutions and serial dilutions were prepared in acetone and stored at 4 °C. Each bioassay used 30 late-third or early-fourth instars placed in plastic cups with 199 mL of dechlorinated water and 1 mL of insecticide solution at the required concentration. We used 5–10 concentrations with three replicates per concentration and 30 larvae per replicate. There were also 3 replicates with 30 larvae each as a blank control, which received only 1 mL of acetone. Tests were performed at 26 ± 1 °C, and mortality was assessed after 24 h of insecticide exposure.

The procedure used for adult bioassays followed the adult mosquito exposure method of the sensitive baseline method recommended by the GB/T 26347-2010 protocol [29]. Papers were impregnated with acetone solutions of insecticide at concentrations ranging from 10 to 160 mg/L, with silicone oil as the carrier. A baseline assay was conducted with three replicates per concentration, using 30 adult female mosquitoes of the reference strain per replicate to determine the LC_99_. The blank control also had three replicates, each containing 30 female adult mosquitoes. The double of the LC_99_ was then used as the diagnostic dose for the field populations.

Three batches of 30 non-blood-fed females (3–5 days old) from each field population were introduced into exposure tubes impregnated with filter paper containing the diagnostic dose of beta-cypermethrin for 60 min. The mosquitoes were then transferred to a recovery tube containing an 8% glucose solution and maintained at 26 ± 1 °C with 65 ± 10% relative humidity. Mortality was recorded 24 h after exposure. For each population, a batch of 30 mosquitoes was used as a control.

### 2.3. Biochemical Assays and Kdr Mutation Detection

Biochemical tests were performed on individual larvae from the same batch for each site population, along with the susceptible strain. The amount of mixed functional oxidase (MFO) was quantified in 30 individual larvae emerging from the 3rd–late or 4th instars. These tests were performed according to adaptations to the instructions from the Centers for Disease Control (CDC) and the WHO [30,31].

PCR amplifications were undertaken for larval mosquitoes (the 3rd–late or 4th instars). Eleven pairs of specific primers for the sodium channel gene described in the article by Li et al. were used [19]. Under PCR amplification conditions, the full-length sequence of the sodium channel gene from *Ae. aegypti* was amplified. The PCR-amplified products were then subjected to electrophoresis on a 1.2% agarose gel, stained with Gold View, and visualized in an ultra-gel documentation system, after which the products were subjected to bidirectional Sanger sequencing by Sangon Biotech (Shanghai, China).

### 2.4. Data Analysis

The Schoofs and Willhite probit analysis program [32] was used to calculate the LC_50_ for the larval assays and the LC_99_ for the adult assay. The resistance ratios (RR) of each population compared to those of the Yunnan strain of *Ae. aegypti* (used as a reference) were calculated as follows: RR = LC_50_ assay/LC_50_ reference. An RR greater than 20 was regarded as high resistance, between 10 and 20 was regarded as moderate resistance, between 2 and 10 was regarded as low resistance, and less than 2 was regarded as susceptibility [33]. For adult bioassays, the resistance or susceptibility status was defined according to GB/T 26347-2010 [29]. Mosquitoes were considered susceptible if the mortality rate was greater than 98% and resistant if the mortality rate was less than 80%. Mortality rates between 80 and 98% suggested possible resistance.

The absorbance values were obtained for the larvae and corrected in relation to the final volume of the larval homogenate, the enzyme activity unit, and the total protein content of each larval pool. Enzyme activity results were determined by comparing the median value for the sensitive strain and five field populations using the Kruskal-Wallis nonparametric test.

The nucleotide sequences of the field populations and susceptible strains were compared using Chromas 2.2. The allele type and genotype were determined, and the VGSC gene allele number and genotype frequency of the samples were determined and calculated. The susceptible homozygote is defined as SS, the mutant heterozygote is defined as RS, and the mutant homozygote is defined as RR; they were calculated as follows:

SS% = SS/(SS + RS + RR) × 100%

RS% = RS/(SS + RS + RR) × 100%

RR% = RR/(SS + RS + RR) × 100%

R% = RR% + 0.5 × RS%

## 3. Results

### 3.1. Larval Bioassay

The insecticide selected for this study was beta-cypermethrin, which is commonly used among the pyrethroids. The sensitive strain of *Ae. aegypti* was used as a reference. The sensitivity baseline method was used to determine the resistance of larvae in five wild populations of *Ae. aegypti* in Yunnan Province. The results are shown in the table below. The LC_50_ of the sensitive strain to the insecticide beta-cypermethrin was 0.00102 mg/L, while the RR of resistance to beta-cypermethrin ranged from 11.31 to 41.56 in the five field populations (Table 2). The five populations of *Ae. aegypti* in Yunnan displayed moderate and high resistance levels.

### 3.2. Establishment of a Diagnostic Dose for Adult Aedes aegypti and Bioassays

#### 3.2.1. Establishment of a Diagnostic Dose for Adult *Aedes aegypti*

The sensitive baseline method was used to establish the diagnostic dose for beta-cypermethrin. The LC_99_ of beta-cypermethrin for adult *Ae. aegypti* mosquitoes was 746.68 mg/L, with a 95% confidence interval of 457–1507. The regression equation was y = −2.7273 + 1.7589x, χ^2^ = 6.167, *p* = 0.940. The diagnostic dose was 2L_99_ = 1493.37 mg/L. The calculated LC_99_ (746.68 mg/L) exceeds the maximum tested concentration (160 mg/L) and is an extrapolation from the probit model. This extrapolation increases uncertainty, reflected in the wide confidence interval (457–1507 mg/L).

#### 3.2.2. Bioassays of Adult *Ae. aegypti*

The results for adult *Ae. aegypti* mortality in the five field populations after treatment with the diagnostic dose of beta-cypermethrin are shown in the table below. The mortality rates of the five field populations ranged from 8.89% to 58.89%, and according to the criteria for judgment, all the field populations were categorized as resistant populations (Table 3).

### 3.3. Correlation Analysis of the Resistance of Larval and Adult Aedes aegypti to Beta-Cypermethrin

**Figure 2:**
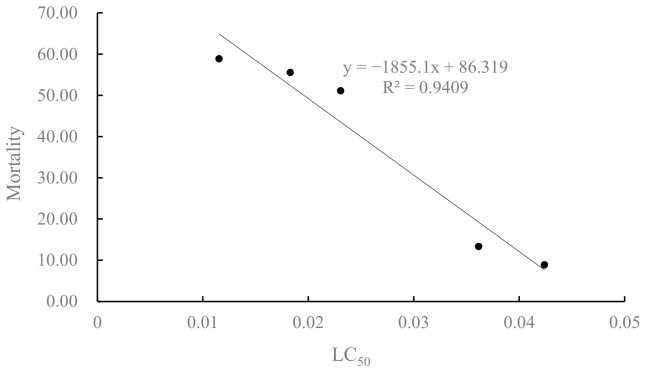
Analysis of the correlation between the LC_50_ of the larvae and the mortality rate of adult *Aedes aegypti* in response to beta-cypermethrin.

The results from the bioassays using adult *Ae. aegypti* were essentially consistent with those of the larval bioassay. Correlations between the mortality rate of adult *Ae. aegypti* at the diagnostic dose and the LC_50_ of the larvae were determined. The results showed a significant linear correlation between the two (R^2^ = 0.9409, *p* = 0.006 < 0.01) (Figure 2).

### 3.4. Multifunctional Oxidase Activity in Larvae

**Figure 3:**
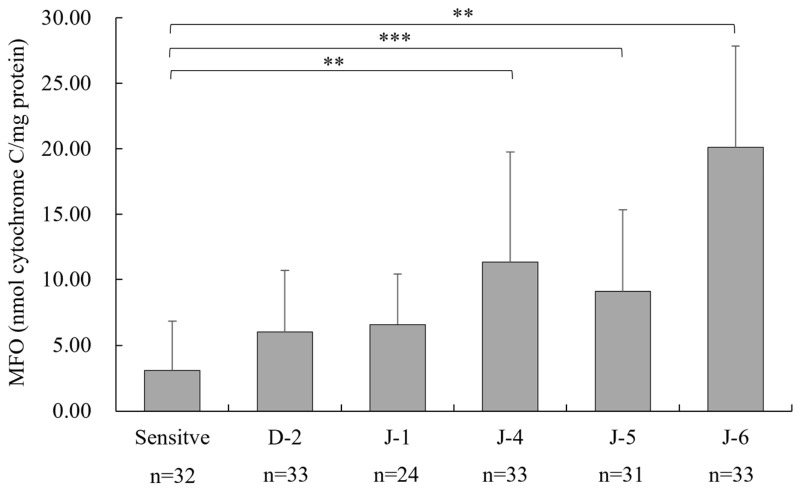
Comparison of MFO amounts in *Aedes aegypti* between the field populations and the sensitive strain. “**” indicates *p* < 0.01; “***” indicates *p* < 0.001.

The MFO amount of the sensitive strain of *Ae. aegypti* was 3.08 ± 3.77 nmol cytochrome C/mg protein, and the MFO amount of all the field populations ranged from 6.01 ± 4.69 to 20.11 ± 7.74 nmol cytochrome C/mg protein. The amount of enzyme in all these populations was greater than that of the sensitive strain (Figure 3). However, only J-4, J-5, and J-6 showed statistically significant differences in enzyme amount compared with the sensitive strain, with *p* values of 0.00117, 0.00016, and 0.00125, respectively.

### 3.5. Types and Frequency of VGSC Gene Mutations

A comparison of the sequencing results with the reference sequence registered in GenBank (No. EU399179.1) revealed a mutation in the amino acid at the 1534 locus from F to C, and the corresponding TTC codon was mutated to TGC. No other mutation loci were identified. The mutation frequencies at the F1534C locus in five field populations of *Ae. aegypti* are shown in Table 4 and ranged from 3.33% to 41.67%.

### 3.6. Correlation Between the F1534C Mutation Frequency and Beta-Cypermethrin Resistance in Aedes aegypti

**Figure 4:**
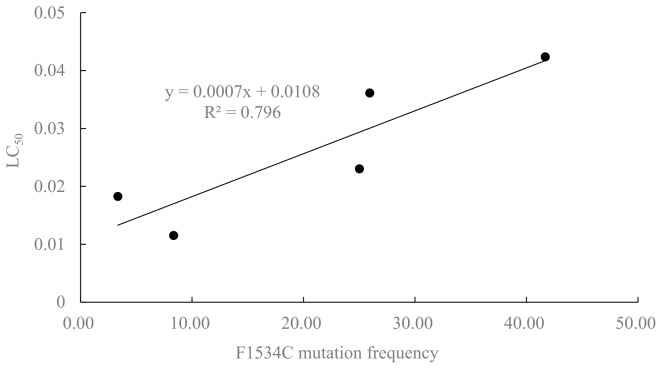
Correlation analysis of the F1534C mutation frequency and the LC_50_ of beta-cypermethrin in *Aedes aegypti*.

The R^2^ and *p* values of the correlation between the F1534C mutation frequency and the mortality of beta-cypermethrin were 0.796 and 0.042, respectively. The data in Figure 4 show a significant positive correlation: the degree of insecticide resistance increased with increasing mutation frequency.

## 4. Discussion

*Aedes aegypti* in Yunnan is a mosquito species imported from Southeast Asia at the beginning of this century, and it invaded by land and water, after which it spread rapidly along the border area. Its distribution area is consistent with the local dengue fever-endemic areas in Yunnan Province, such as Ruili in Dehong and Jinghong in Xishuangbanna, where *Ae. aegypti* is the dominant species in the main urban area, and the composition ratio is significantly higher than that of *Ae. albopictus* [34]. *Ae. aegypti* has been the main vector of dengue fever in the China–Myanmar and China–Laos border areas in Yunnan Province in recent years. Therefore, chemical control of the vector *Ae. aegypti* is essential for the regional prevention and control of dengue fever. However, the overapplication and inappropriate use of insecticides have led to the selection of resistance in *Ae. aegypti*, resulting in a decrease in the effectiveness of related control measures [35]. Elucidating the status of resistance to commonly used insecticides in local *Ae. aegypti* populations is urgently needed to develop effective strategies to manage resistance [36].

Bioassay results for beta-cypermethrin resistance in *Ae. aegypti* from Yunnan Province, China, indicated that the RR of the five populations ranged from 11.31 to 41.56, indicating moderate to high levels of resistance to beta-cypermethrin. Research has indicated that in 2006, approximately 52.63% of household insecticides in China were pyrethroid insecticides [37], and by 2009, this percentage had increased to 84.45% [38]. This shift may have occurred because *Ae. aegypti* typically breeds indoors or around dwellings. In China’s public health market, pyrethroid insecticides are widely used in indoor sprays or incense or are applied to mosquito nets, curtains, and window screens. Since 2013, Xishuangbanna has frequently experienced dengue fever outbreaks, and pyrethroid insecticides are commonly used for vector control of *Aedes* mosquitoes, such as residual spraying and space spraying [39].

*Aedes aegypti* is a common domestic species found in small water containers, and it has been chronically exposed to various insecticides used in forestry and other sectors [33]. Additionally, water pollution caused by pyrethroid insecticides used in agricultural activities may also contribute to the development of resistance [40]. The results of the bioassay for adult *Ae. aegypti* were consistent with the larval resistance findings. This study established a diagnostic dose of beta-cypermethrin for adult *Ae. aegypti*; although the process was complex and labor-intensive, it serves as a valuable reference standard for conducting resistance testing and guiding the rational use of insecticides. Compared with previous studies that directly used published diagnostic doses, this study established a local diagnostic dose based on Yunnan’s susceptible strain, which improves the accuracy and applicability of resistance monitoring in local *Ae. aegypti* populations.

In studies on insecticide resistance and metabolic enzyme activity in mosquitoes, the most common enzymes involved in metabolic detoxification related to pyrethroid resistance include MFO and esterases [12]. Among these enzymes, MFO is an oxidase system comprising cytochrome P450, cytochrome b5, NADPH cytochrome P450 reductase, and other proteins. Cytochrome P450 is the most critical component and is involved in the metabolic detoxification of various endogenous and exogenous substances [15]. Elevated MFO levels increase metabolic detoxification, constituting one of the primary mechanisms through which mosquitoes develop metabolic resistance to pyrethroid insecticides [41].

Compared with that of the susceptible strain, the MFO amount of all five field populations of *Ae. aegypti* tested in this study was higher, and three populations showed significant differences. This result may be one reason for the increased tolerance to beta-cypermethrin observed in wild *Ae. aegypti* populations in Yunnan. Biochemical studies on wild *Ae. aegypti* populations in Trinidad and Tobago, Central America, have shown an association between increased MFO amount and resistance to DDT and pyrethroids [42]. Furthermore, studies have indicated that among eight geographically distinct *Ae. aegypti* populations collected from Thailand, MFO enzyme activity was significantly increased in six pyrethroid-resistant samples [43]. A biochemical analysis of *Ae. aegypti* populations in Indonesia by Amelia-Yap et al. revealed that elevated MFO amount is correlated with the pyrethroid resistance phenotype, indicating that MFO plays a crucial role in this resistance mechanism [44].

Overall, the pyrethroid resistance mechanism in *Ae. aegypti* from Yunnan, China, is partially conferred by increased MFO amount. Moreover, insecticide resistance arises from multiple factors, including metabolic detoxification and target gene mutations. Therefore, this study further examined alterations in the VGSC gene of *Ae. aegypti*. The results indicated that only the 1534 locus exhibited amino acid mutations. A correlation analysis with the pyrethroid resistance phenotype revealed that an increased frequency of F→C mutations increased resistance in *Ae. aegypti*. Previous studies have demonstrated that the mutations V410L, S989P, I1011M, V1016G, and F1534C in *Ae. aegypti* increase insect resistance to pyrethroids [22,45]. Furthermore, studies indicate that specific double mutations or triple mutations in *Ae. aegypti* exert synergistic effects, further reducing sensitivity to pyrethroids. Examples include the S989P + V1016G double mutations found in Indonesia [46], the V1016G + F1534C double mutations found in Laos [47], the T1520I + F1534C double mutations found in Pakistan [48], and the S989P + V1016G + F1534C triple mutations found in Thailand [49].

The F1534C mutation and increased MFO enzyme activity may jointly alter the resistance of *Ae. aegypti* in Yunnan to pyrethroid insecticides. This finding shares a similar mechanism with studies conducted in different regions: permethrin resistance in the Niamey *Ae. aegypti* population in Africa is underpinned by the triple mutations 1534CC + 1016LL + 410LL and potentially accompanied by increased esterase activity [50]. The F1534C, V1016G, and S989P mutations significantly promoted pyrethroid resistance in the Malaysian *Ae. aegypti* population, with the MFO and GST enzymes playing partial roles [12].

This study has several limitations, including the limited number of sampling sites and insecticides used in the experiments. Our sampling was limited to two prefectures in Yunnan; findings may not represent *Ae. aegypti* resistance across the entire province. Our data were collected in 2015–2016; resistance levels may have changed since then, and no confirmatory bioassays were performed by us after 2020. Further research is needed to investigate the spatial distribution of insecticide resistance in *Ae. aegypti* mosquitoes in Yunnan and to determine whether similar resistance mechanisms apply to a broader range of insecticides.

*Aedes aegypti* possesses strong adaptability and reproductive capacity. Under intense control pressure from the extensive use of insecticides targeting mosquito-borne diseases, it has undergone various genetic mutations. Global climate change and urbanization have created favorable conditions for the spread of *Ae. aegypti*, while genes associated with pyrethroid resistance in this mosquito species are rapidly spreading across Yunnan Province. Research data from different periods indicate that in the population sampled by Li et al. in Yunnan in 2013, the F1534C mutation frequency was relatively low (4.6–7.6%), while the double mutation S989P + V1016G occurred at a frequency of 66.7–100%. No triple mutations were detected [19]. In samples of *Ae. aegypti* collected from 2015 to 2016 in this study, the mortality rates of beta-cypermethrin at the diagnostic dose ranged from 8.89% to 58.89%, and the mutation frequency of F1534C was measured to be 3.33–41.67%. Wang et al. reported that in *Ae. aegypti* populations collected in Yunnan during 2019–2020, the mortality rates of permethrin and beta-cyfluthrin at the diagnostic dose were 0–65.56% and 5.77–77.17%, respectively, indicating that these populations were resistant. The frequency of the F1534C mutation rose to 21.7–94.2%, and the double mutations V1016G + F1534C and S989P + V1016G were detected [35]. Recently, Chen et al. detected that in *Aedes aegypti* collected along the Yunnan–Myanmar border in 2022, the mortality rates of permethrin and lambda–cyhalothrin at diagnostic doses were 18.1–21.9% and 11.4–36.1%, respectively; the frequencies of the F1534C, S989P, and V1016G mutations were 78.9%, 81.6%, and 100%, respectively; and the detection rate of the S989P + V1016G + F1534C triple mutation reached 60.8% [34]. In 2024, a study on *Ae. aegypti* collected in Xishuangbanna, Yunnan, found mortality rates as low as 1.69–7.46% and 0.98–16.92% at diagnostic doses of permethrin and lambda–cyhalothrin, respectively, indicating that the level of resistance has reached an extremely high level [51]. These findings indicate that pyrethroid resistance and the associated genes have spread rapidly among *Ae. aegypti* populations of Yunnan in recent years.

Therefore, we recommend that local public health systems implement continuous surveillance of insecticide resistance in *Ae. aegypti* populations to inform practical decision-making and formulate policies for vector-borne disease control measures. First, based on the high resistance of *Ae. aegypti* to pyrethroid insecticides and the high frequency of F1534C mutation in the study area, it is recommended to avoid the long-term single use of pyrethroid insecticides to delay the further development of resistance. Second, it is recommended to rotate pyrethroids with other insecticides with different mechanisms of action (such as organophosphates) to reduce the selection pressure of resistance. Third, the F1534C mutation can be used as a molecular marker for monitoring pyrethroid resistance in field populations of *Ae. aegypti*, providing a scientific basis for public health departments to adjust vector control strategies.

## Figures and Tables

**Table 1:** Detailed information on field sampling sites in Yunnan Province in 2015–2016.

Collection Region	Code	Location Name	Geographical Coordinates	Breeding Site Types	Sampling Area
Xishuangbanna Prefecture	J1	Jingha Township	21°50′01″ N 100°55′49″ E	small water-holding containers such as bottles and jars, discarded tires, tree holes	1 km^2^
	J4	Ganlanba	21°51′26″ N 100°55′37″ E	small water-holding containers such as bottles and jars, discarded tires, tree holes	1 km^2^
	J5	Xishuangbanna Tropical Botanical Garden	21°56′25″ N 101°15′41″ E	small water-holding containers such as bottles and jars, discarded tires, tree holes	1 km^2^
	J6	Gasa Township	21°57′05″ N 100°45′44″ E	small water-holding containers such as bottles and jars, discarded tires, tree holes	1 km^2^
Dehong Prefecture	D2	Jie’gao Freight Yard	23°58′40″ N 097°53′24″ E	small water-holding containers such as bottles and jars, discarded tires, tree holes	1 km^2^

**Table 2:** Resistance to beta-cypermethrin among five field populations of *Aedes aegypti* larvae in Yunnan Province.

Population	LC_50_ (mg/L)	95% CI	Regression Equation	χ^2^	*p*	RR
Sensitive	0.00102	0.00088–0.00118	y = 5.33870 + 1.78533x	6.867	0.995	1
D-2	0.01154	0.00949–0.01336	y = 4.92405 + 2.54126x	20.577	0.082	11.31
J-1	0.02306	0.02076–0.02528	y = 5.13257 + 3.13522x	21.827	0.293	22.61
J-4	0.03615	0.03363–0.03873	y = 5.54634 + 3.84644x	13.808	0.795	35.44
J-5	0.01829	0.01679–0.01976	y = 8.42712 + 4.84928x	9.018	0.772	17.93
J-6	0.04239	0.03931–0.04566	y = 4.92428 + 3.58724x	13.159	0.661	41.56

LC_50_ = median lethal concentration; CI: confidence interval; χ^2^: Chi-square value; *p*: *p*-value; RR = resistance ratio.

**Table 3:** Resistance to beta-cypermethrin among five field populations of adult *Aedes aegypti* in Yunnan Province.

Population	Total	Number of Deaths	Mortality (%)	Degree
Sensitive	90	90	100.00	susceptible
D-2	90	53	58.89	resistant
J-1	90	46	51.11	resistant
J-4	90	12	13.33	resistant
J-5	90	50	55.56	resistant
J-6	90	8	8.89	resistant

Degree: >98%, susceptible; 80–98%, possible resistance; <80%, resistant.

**Table 4:** F1534C mutation frequency in the VGSC gene of *Aedes aegypti*.

Population	N	Genotype and Gene Frequency			
		SS	RS	RR	R (%)
D-2	30	26	3	1	8.33
J-1	30	16	13	1	25.00
J-4	27	15	10	2	25.93
J-5	30	28	2	0	3.33
J-6	30	7	21	2	41.67

## Data Availability

The original contributions presented in this study are included in the article. Further inquiries can be directed to the corresponding author.

## Abbreviations

The following abbreviations are used in this manuscript:

MFO	Mixed-function oxidase
VGSC	Voltage-gated sodium channel
PCR	Polymerase chain reaction
CDC	Centers for Disease Control
WHO	World Health Organization
